# New ESC guidelines on hypertrophic cardiomyopathy: new insights in invasive treatment?

**DOI:** 10.1007/s12471-014-0636-7

**Published:** 2014-12-05

**Authors:** E. E. van der Wall

**Affiliations:** Interuniversity Cardiology Institute of the Netherlands (ICIN) - Netherlands Heart Institute (NHI), PO Box 19258, 3501 DG Utrecht, the Netherlands

Hypertrophic cardiomyopathy (HCM) is a complex, yet relatively common genetic cardiac disease and has been the subject of intensive investigation since its first description in 1958. HCM is defined by the presence of an increased left ventricular wall thickness that is not solely explained by abnormal loading conditions. Histologically, HCM is characterised as left ventricular hypertrophy due to an abnormally hypertrophied muscular structure of predominantly the septum (‘myocardial fibre disarray’) [1]. Approximately 30 % of patients with HCM develop left ventricular outflow tract (LVOT) obstruction under resting conditions [2–4]. By convention, LVOT obstruction is defined as an instantaneous peak Doppler LVOT gradient of > 30 mmHg at rest or during physiological provocation. A gradient of > 50 mmHg is usually considered to be the threshold at which LVOT obstruction becomes haemodynamically significant.

Pharmacological therapy using negative inotropic agents (non-vasodilating beta-blocking agents, calcium antagonists in particular verapamil, and disopyramide) is effective in the majority of patients. However, 5–10 % of patients experience drug-refractory symptoms most likely due to an increased LVOT gradient. In those patients, surgical septal myectomy (Morrow procedure) has been advocated to reduce outflow obstruction and relieve symptoms. However, over the years there have been many controversies over the efficacy of myectomy as there were, and still are, serious doubts about the haemodynamic impact of the septal obstruction. Is HCM due to an abnormal ejection or due to an abnormal filling of the left ventricle; in other words, is HCM primarily a systolic or a diastolic problem? On one side, it was Maron and Braunwald (Minneapolis, Boston) who emphasised the importance of the obstruction [5], on the other hand, Criley (UCLA, California) and Murgo (San Antonio, Texas) defended the filling theory [6, 7]. Still in the year 2010 Murgo wrote the following words in JACC [6]: *there is no evidence that outflow is compromised as a result of an LVOT gradient. Such an understanding does not imply that elimination of LVOT gradients is not potentially beneficial. Rather, one hopes that when one does recommend an intervention to eliminate such gradients, one understands that that intervention is not designed to improve ejection itself*. Of course this was contradicted by Maron and Braunwald, who supported the obstruction theory and therefore advocated the surgical approach. It is also of interest to take notice of the paper by Maron et al. [8], published in 2011(!) in the European Heart Journal (EHJ), where they encouraged the European cardiology and cardiothoracic-surgery community to reconsider surgical septal myectomy as a treatment option for severely symptomatic obstructive HCM patients within Europe. This was based on the fact that the less invasive alcohol septal ablation, introduced by Ulrich Sigwart (Geneva, London) in 1994, became more and more common practice in Europe resulting in the virtual obliteration of the surgical option for HCM patients in Europe [9].

What do the new ESC guidelines, published in the EHJ October 2014 issue [10], teach us about the value of invasive therapy in severely affected HCM patients? What is the current status of myectomy, septal alcohol ablation and dual chamber pacing? First of all, there are no data to support the use of invasive procedures to reduce LVOT gradients in asymptomatic patients, regardless of their severity. In symptomatic patients, however, with LVOT gradients of > 50 %, surgical myectomy substantially reduces the LVOT gradient in over 90 % of patients whereby long-term symptomatic benefit is achieved in 70–80 % of patients with a long-term survival comparable with that of the general population. In those patients myectomy has a Class I recommendation signifying that the procedure is recommended/indicated. The main surgical complications are AV-nodal block, ventricular septal defect and aortic regurgitation, but these complications are uncommon in experienced centres using intraoperative transoesophageal echocardiography guidance. Surgical mortality for myectomy with mitral intervention is 3–4 %.

Regarding the value of alcohol septal ablation, there are no randomised trials comparing surgery and alcohol septal ablation but several meta-analyses have shown that both procedures improve functional status with similar procedural mortality [11]. Alcohol septal ablation is the preferred option in patients with mid-ventricular obstruction and appropriate coronary anatomy [12]. However, the less invasive version of the septal reduction therapy comes at a price: a larger number of right and left bundle branch blocks, a higher risk on total AV blocks (7–20 %) requiring permanent pacemaker implantations, and larger residual LVOT gradients [13]. The new guidelines therefore state that septal myectomy, rather that alcohol septal ablation, is recommended (Class I) in patients with an indication for septal reduction therapy who also have other lesions requiring surgical interventions (lesions of valves and papillary muscles). It is also imperative that, because of the variability of the septal blood supply, myocardial contrast echocardiography is essential prior to alcohol injection. If, for instance, the contrast agent cannot be localised exclusively to the basal septum, the procedure should be abandoned. Recently, septal myocardial ablation using microsphere embolisation was proposed as an alternative to alcohol to treat patients with HCM [14].

Regarding the value of dual-chamber pacing, only three small randomised placebo-controlled studies have reported reductions in LVOT obstruction gradients but variable improvements in symptoms and quality of life [15]. A recent Cochrane review concluded that the data on the benefits of pacing are based on physiological measures and they lack information on clinically relevant endpoints. As a result, cardiac pacing in HCM has a Class IIb recommendation implying that the usefulness/efficacy of the treatment/procedure is less well established by evidence/opinion.

To summarise, the authors of the new HCM guideline have stayed away from the controversy between LVOT obstruction versus left ventricular emptying as the most important functional parameter in HCM patients. Obviously, they have implicitly chosen for the LVOT gradient as the key determinant in HCM. Consequently, the new guidelines do support the use of septal reduction therapy, i.e. myectomy and alcohol septal ablation, in symptomatic patients with an LVOT gradient > 50 mmHg provided that the septal reduction therapy is performed by experienced operators working as a part of a multidisciplinary team expert in the management of HCM. With these precautions in mind -in particular for alcohol septal ablation- these invasive procedures have a Class I recommendation, indicating that there is nowadays sufficient evidence and/or general agreement that these procedures are beneficial, useful, and effective. However, experienced hands are a conditio sine qua non.
